# Comprehensive identification and characterization of lncRNAs and circRNAs reveal potential brown planthopper-responsive ceRNA networks in rice

**DOI:** 10.3389/fpls.2023.1242089

**Published:** 2023-08-10

**Authors:** Yan Wu, Wenjun Zha, Dongfeng Qiu, Jianping Guo, Gang Liu, Changyan Li, Bian Wu, Sanhe Li, Junxiao Chen, Liang Hu, Shaojie Shi, Lei Zhou, Zaijun Zhang, Bo Du, Aiqing You

**Affiliations:** ^1^ Key Laboratory of Crop Molecular Breeding, Ministry of Agriculture and Rural Affairs, Hubei Key Laboratory of Food Crop Germplasm and Genetic Improvement, Institute of Food Crops, Hubei Academy of Agricultural Sciences, Wuhan, China; ^2^ Hubei Hongshan Laboratory, Wuhan, China; ^3^ State Key Laboratory of Hybrid Rice, College of Life Sciences, Wuhan University, Wuhan, China

**Keywords:** rice, brown planthopper, long non-coding RNA, circular RNA, competitive endogenous RNA

## Abstract

Brown planthopper (*Nilaparvata lugens* Stål, BPH) is one of the most destructive pests of rice. Non-coding RNA plays an important regulatory role in various biological processes. However, comprehensive identification and characterization of long non-coding RNAs (lncRNAs) and circular RNAs (circRNAs) in BPH-infested rice have not been performed. Here, we performed a genome-wide analysis of lncRNAs and circRNAs in *BPH6*-transgenic (resistant, BPH6G) and Nipponbare (susceptible, NIP) rice plants before and after BPH feeding (early and late stage) via deep RNA-sequencing. A total of 310 lncRNAs and 129 circRNAs were found to be differentially expressed. To reveal the different responses of resistant and susceptible rice to BPH herbivory, the potential functions of these lncRNAs and circRNAs as competitive endogenous RNAs (ceRNAs) were predicted and investigated using Gene Ontology and Kyoto Encyclopedia of Genes and Genomes analyses. Dual-luciferase reporter assays revealed that miR1846c and miR530 were targeted by the lncRNAs XLOC_042442 and XLOC_028297, respectively. In responsive to BPH infestation, 39 lncRNAs and 21 circRNAs were predicted to combine with 133 common miRNAs and compete for miRNA binding sites with 834 mRNAs. These mRNAs predictably participated in cell wall organization or biogenesis, developmental growth, single-organism cellular process, and the response to stress. This study comprehensively identified and characterized lncRNAs and circRNAs, and integrated their potential ceRNA functions, to reveal the rice BPH-resistance network. These results lay a foundation for further study on the functions of lncRNAs and circRNAs in the rice-BPH interaction, and enriched our understanding of the BPH-resistance response in rice.

## Introduction

Rice, one of the most important food crops worldwide, is host to more than 200 insect pests at different stages of its life cycle. Among these, the brown planthopper (*Nilaparvata lugens* Stål, BPH) is one of the most destructive. Recent surveys suggest that BPH damages rice production severely, accounting for 29.5% of the total rice crop loss due to insects and diseases and making BPH the number one pest of Chinese rice (Du et al., 2020). Therefore, the development and production of BPH-resistant rice varieties is an economical, effective, safe, and environmentally-friendly strategy to control BPH damage (Yu et al., 2022).

In order to improve the resistance of rice to BPH, the genes and signaling pathways pivotal to BPH resistance must be identified and characterized. Over the last five decades, around 40 BPH resistance genes (*R* genes) have been identified, and the mechanisms of rice resistance to BPH have been explored through genetic and biochemical analyses (Chen et al., 2022; Guo et al., 2023). Recently, the novel BPH *R* gene *BPH6*, encoding an exocyst-localized protein, was cloned and found to confer broad-spectrum resistance to planthoppers (Guo et al., 2018). Specifically, the *BPH6* gene promotes exocytosis and participates in the maintenance and reinforcement of plant cell wall (Wu et al., 2022). Further functional characterization of *BPH6* will more precisely illuminate the exact mechanism underpinning *BPH6*-mediated herbivory resistance.

microRNAs (miRNAs) have been demonstrated to regulate rice resistance to various pathogens and herbivores by modulating the expression of target genes at the post-transcriptional level (Kar and Raichaudhuri, 2021). For example, both miR159 and miR160 regulate the defense response against blast disease (Li et al., 2014; Chen et al., 2021), and miR159b, miR164a, and miR167d-5p are involved in the plant immune response to bacterial blight (Jia et al., 2020). These miRNAs fine-tune plant innate immunity through the integration of *R* gene expression, phytohormone signaling, callose deposition, and reactive oxygen species (ROS) production (Kumar et al., 2022). Two miRNAs (miR156 and miR396) have been reported to regulate rice resistance to BPH. Specifically, miR156 regulates jasmonic acid (JA) and jasmonoyl-isoleucine (JA-Ile) biosynthesis through the “miR156-*OsMPK3/6*-*OsWRKY70*” module, thus negatively regulating BPH resistance (Ge et al., 2018). miR396 also negatively regulates BPH resistance through the “miR396-*OsGRF8*-*OsF3H-*flavonoid” module (Dai et al., 2019).

Recent studies revealed that long non-coding RNAs (lncRNAs) and circular RNAs (circRNAs) may serve as competing endogenous RNAs (ceRNAs), which could be essential for regulating the circuitry of miRNAs and their target genes (Salmena et al., 2011). Specifically, lncRNAs and circRNAs can modulate the balance between miRNAs and target genes. lncRNAs are eukaryotic non-coding RNAs greater than 200 nucleotides (nt) in length (Kapranov et al., 2007; Ponjavic et al., 2007). They have been found to regulate both biotic and abiotic stress tolerance, as well as various growth and developmental processes in rice (Gao et al., 2020). Non-coding endogenous circRNA molecules are covalently closed continuous loops without 5’-3’ polarity or a polyadenylated tail and have been found to respond to abiotic and biotic stimuli and growth processes (Jeck et al., 2013). However, the specific regulatory mechanism underlying lncRNA- and circRNA-mediated BPH resistance in rice remains to be elucidated.

In this study, we performed a genome-wide analysis of lncRNAs and circRNAs in *BPH6*-transgenic (resistant, BPH6G) and Nipponbare (susceptible, NIP) rice plants before and after BPH herbivory via deep RNA-sequencing. We also conducted an integrated analysis of the expression profiles of circRNAs, lncRNAs, and previously identified miRNAs and mRNAs (Tan et al., 2020). lncRNA/circRNA-miRNA-mRNA ceRNA networks were generated by combining the identified and annotated target mRNAs. Our results demonstrated that lncRNAs and circRNAs act as ceRNAs to regulate BPH resistance in rice. This study provides a foundation for further research into the molecular mechanisms underlying *BPH6*-conferred herbivory resistance in rice.

## Materials and methods

### Plant and BPH materials

Two rice lines were used in this study: Nipponbare (NIP) and *BPH6*-transgenic plants (BPH6G). NIP is a susceptible *japonica* line. BPH6G is a *BPH6*-transgenic line containing the *BPH6* gene with its native Swarnalata promoter (IRRI Acc. No. 33964) in NIP background. The rice plants were grown in plastic cups (9 cm in diameter and 15 cm in height), in a greenhouse with 32 ± 2°C/14 h light and 26 ± 2°C/10 h dark periods. The BPH population was maintained on the susceptible rice cv. Taichung Native1 (IRRI Acc. No.00105) under controlled environmental conditions (as described above) at Wuhan University (Hu et al., 2017).

### Evaluation of rice resistance to BPH

Seeds of either NIP or BPH6G were sown in plastic pots covered with nylon mesh. At the four-leaf stage, the rice seedlings were infested with third instar BPH nymphs at a rate of 10 nymphs per plant. Observation continued until susceptible control plants died, and then the rice plants were subsequently photographed and scored. At least three independent biological replicates were performed.

Measurements of honeydew excretion and BPH weight gain were performed following the method of Zheng et al. (Zheng et al., 2021). Twenty female BPH adults, which had been starved for 2 h, were weighed and introduced into a pre-weighed parafilm sachet (1.5 cm × 2.5 cm) fixed on the leaf sheath of each five-leaf stage NIP or BPH6G plant at a height of 2-3 cm above the soil. After 48 h of feeding, the surviving insects and the parafilm sachet were weighed again. The change in the insects’ weight was recorded as BPH weight gain, and the change in the sachets’ weight was recorded as honeydew excretion.

### Sample collection

The endpoint method was utilized for sample collection throughout BPH treatment (Wu et al., 2017). Each treatment began at an individual time point and stopped at the same time. Seedlings at the four-leaf stage were infested with eight BPHs per seedling and collected after 0, 6, 12, 24, 48, 60, and 72 hours of infestation. Each treatment consisted of three biological replicates, with 15 seedlings per replicate. Leaf sheaths were mixed for non-infested controls (0 h), early infestation stage (6, 12, and 24 h), and late infestation stage (48, 60, and 72 h). BPH6G samples were annotated as R0, R_early, and R_late, and NIP samples were annotated as S0, S_early, and S_late. Leaf sheathes were excised, frozen in liquid nitrogen, and stored at -80°C until use.

### Total RNA extraction and high-throughput sequencing

RNA was isolated using an RNAiso Plus kit (TaKaRa), according to the manufacturer’s instructions. RNA quality was estimated using a NanoDrop 2000 spectrophotometer (Thermo Fisher Scientific). An Illumina HiSeq 2500 was used for total RNA sequencing. The raw reads were first quality-controlled with FAST-QC by filtering low-quality reads (http://www.bioinformatics.babraham.ac.uk/projects/fastqc/). Afterward, clean reads were aligned to the rice genome using Hisat2 software (Kim et al., 2015).

### Identification of lncRNAs and differential expression analysis

The mapped reads were assembled using StringTie (Pertea et al., 2015). All transcriptomes were merged to reconstruct a comprehensive transcriptome using Cuffmerge. Transcripts overlapping with known mRNAs and transcripts shorter than 200 bp were discarded. To identify lncRNAs, we utilized CPAT to predict coding transcripts, and transcripts with coding-prob score > 0.3 were removed (Wang et al., 2013). Transcript expression levels were quantified as fragments per kilobase of exon per million fragments mapped (FPKM). Differential expression *P*-values were calculated using the Bioconductor edgeR package (Robinson et al., 2010). We used the absolute value of log_2_FC ≥ 1 and *P* < 0.05 as thresholds to judge the statistical significance of each differential expression result.

### Identification of circRNAs and differential expression analysis

The sequencing data was used to predict circRNAs with the ACFS circRNA prediction pipeline (You and Conrad, 2016). Unmapped reads were obtained with BWA-MEM for circRNA identification. The head-to-tail junction was identified and the highest splicing strength score was calculated using MaxEntScan33, with a filtering criterion greater than or equal to 10. Based on the re-alignment of the unmapped reads to the circRNA candidates, reads which mapped to the circRNA back splicing junction (with an overhang of at least 6 nucleotides) were counted for each circRNA. Transcript expression levels were quantified as FPKM. Differential expression *P*-values were calculated using the Bioconductor edgeR package (Robinson et al., 2010). We used the absolute value of log_2_FC ≥ 1 and *P* < 0.05 as thresholds to judge the statistical significance of each differential expression result.

### Construction and analysis of the ceRNA regulatory network

Based on ceRNA theory (Salmena et al., 2011), we predicted the miRNA-mRNA, miRNA-lncRNA, and miRNA-circRNA interaction pairs using the PsTarget platform, with the standard E = 5 and UPE = 25 (http://plantgrn.noble.org/psRNATarget/analysis). Subsequently, through a combined analysis of miRNA and mRNA expression, lncRNA/circRNA-miRNA-mRNA pathways exhibiting either up-down-up or down-up-down expression modes were selected for further study. Cytoscape3.9.1 was used to display the networks (Shannon et al., 2003).

### Analysis of Gene Ontology and Kyoto Encyclopedia of Genes and Genomes pathways

GO annotations from Gene Ontology (http://www.geneontology.org/) were downloaded (Ashburner et al., 2000). Pathway annotations were downloaded from KEGG (http://www.genome.jp/Kegg/). To identify significant GO and pathway categories, Fisher’s exact tests were applied under absolute values of *P* < 0.05 and FDR < 0.05 (Draghici et al., 2007).

### Quantitative real-time PCR assay

First-strand cDNA was synthesized with a PrimeScript RT reagent kit (TaKaRa, AK2802) using 1 μg of total RNA. The cDNA was amplified by qRT-PCR using SYBR green supermix (Bio-Rad) and a CFX96 real-time system, according to the manufacturer’s instructions. All qRT-PCR primers used in this work are listed in **Supplementary Table 1**. Three biological replicates were performed for each experiment. Normalized expression levels were calculated using the 2^−ΔΔC (t)^ method, with *TBP* as the internal reference gene.

### Luciferase reporter assays

The wild type (WT) and mutated (MUT) lncRNAs containing target miRNA binding sites were synthesized and cloned into the pGreenII 0800-miRNA reporter vector, yielding the lncRNA-WT and lncRNA-MUT vectors, respectively. The miRNA was amplified and cloned into the pCXUN overexpression vector, yielding the miRNA-OE vector. The miRNA-OE/lncRNA-WT, miRNA-OE/lncRNA-MUT, empty vector (PCXUN)/lncRNA-WT, and empty vector (PCXUN)/lncRNA-MUT pairs were co-transfected into rice protoplast cells, which were prepared following the method of Wu et al. (Wu et al., 2017). After 16 h of transfection, luciferase activities were evaluated with a dual-luciferase reporter assay system (Promega) and a SpectraMax iD5 multi-mode microplate reader (Molecular Devices). In this study, two lncRNA-miRNA interaction pairs (XLOC_042442-miR1846c and XLOC_028297-miR530) were selected to perform the assay **(** **Supplementary Table 1** **)**.

## Results

### lncRNA and circRNA expression in BPH6G and NIP before and after BPH feeding

**Figure 1:**
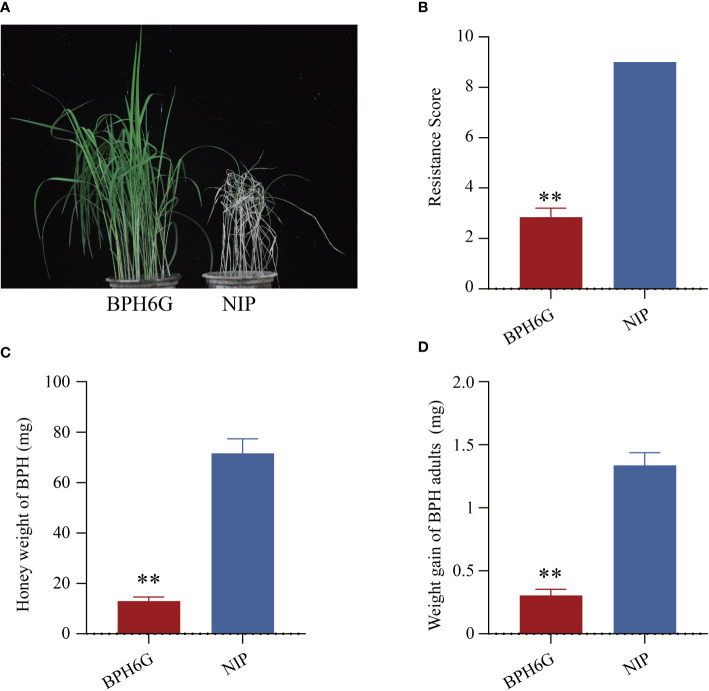
Evaluation of BPH resistance in BPH6G and NIP plants. **(A)** BPH resistance phenotypes of BPH6G and NIP plants. **(B)** BPH resistance scores of BPH6G and NIP plants. Lower scores correspond to greater resistance. Data represent the means ± SE of 3 biologically independent experiments. **(C)** Honeydew excretion of BPHs on BPH6G and NIP plants after 2 d of feeding. Data represent the means ± SE of 10 replicates. **(D)** Weight gain of BPHs after 2 d of feeding on BPH6G and NIP plants. Data represent the means ± SE of 10 replicates. Data were analyzed by ANOVA and asterisks indicate statistically significant differences. **P < 0.01.

We evaluated the resistance of *BPH6*-transgenic plants (BPH6G) and susceptible Nipponbare plants (NIP) to BPH at the seedling stage, and the average damage severity score was calculated for each plant after infestation. By the 7th day of BPH infestation, the NIP plants died (average score of 9.0), while the BPH6G plants were still vigorously growing (average score of 2.8) (**Figures 1A, B**). To confirm these results, we measured the honeydew excretion and weight gain of BPHs allowed to feed on NIP and BPH6G plants for 48 h. BPHs on BPH6G plants excreted less honeydew and had a lower growth rate than BPHs on NIP plants (**Figures 1C, D**). These results demonstrate that BPH6G confers resistance to BPH infestation, while NIP remained susceptible.

To identify BPH-responsive non-coding RNAs in BPH6G (R) and NIP (S) plants, we obtained the FPKM values of lncRNAs and circRNAs from the whole-transcriptome RNA sequencing data after 0, 6, 12, 24, 48, 60, and 72 h of BPH infestation. The 0 h samples were taken as the non-infested controls, while the mixtures of 6, 12, and 24 h samples were taken as early-stage profiles and the mixtures of 48, 60, and 72 h samples were taken as late-stage profiles. In total, 6 treatment groups were analyzed: R0, R_early, R_late, S0, S_early, and S_late.

### Analysis of lncRNA characteristics and response to BPH invasion

**Figure 2:**
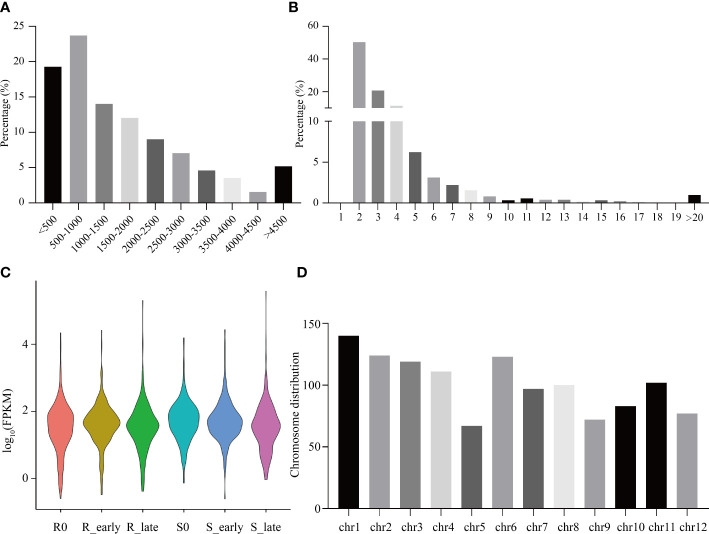
Identification and distribution of all lncRNAs. **(A)** Lengths of lncRNAs. **(B)** Number of exons. **(C)** FPKM distribution of lncRNAs in six groups. **(D)** Chromosomal distribution of all lncRNAs.

The raw reads from 18 rice transcriptomes were combined and filtered, resulting in the identification of 1219 lncRNAs. Most lncRNAs were less than 2,000 nt in length (**Figure 2A**), with the majority containing 2-4 exons, and 50.29% containing two exons (**Figure 2B**). The six treatment groups (R0, R_early, R_late, S0, S_early, and S_late) exhibited different lncRNA expression profiles (**Figure 2C**). The chromosomal distribution of the identified lncRNAs is shown in **Figure 2D**. Different chromosomes contained different numbers of lncRNAs, with chromosome 1 (Chr 1) containing the greatest number (**Figure 2D**).

**Figure 3:**
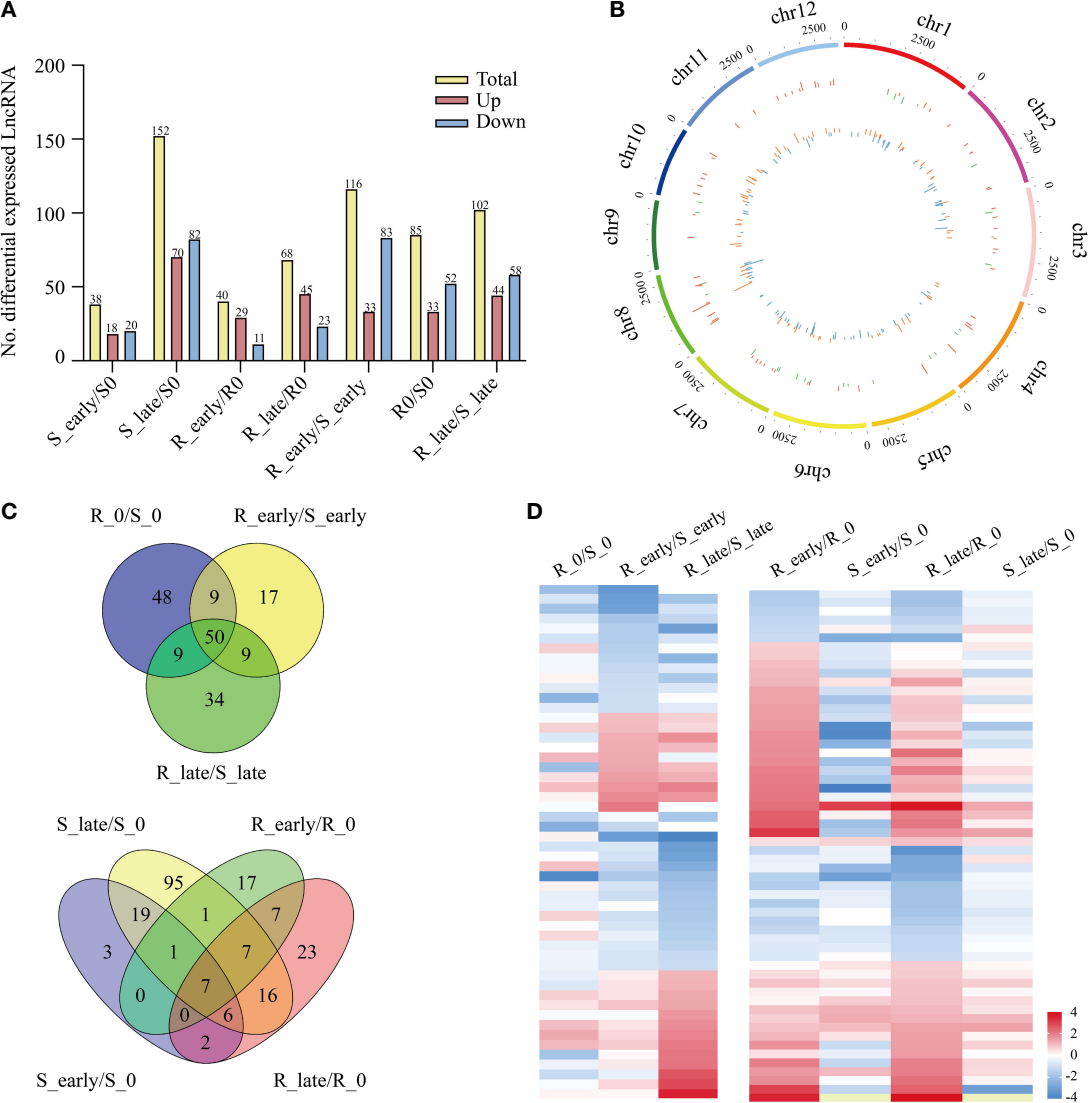
Differentially expressed lncRNAs. **(A)** Number of DElncRNAs up- or down-regulated in all comparisons. **(B)** Genomic distribution of all DElncRNAs. The two circles (from outer to inner) represented the expression levels (log_2_FC) of DElncRNAs in the resistant plants (red indicates increased expression, and green denotes decreased expression), and in the susceptible plants (orange indicates increased expression, and blue denotes decreased expression), and fold change of the expression levels, respectively. **(C)** Venn diagrams of the unique and shared DElncRNAs. **(D)** Heat map of the DElncRNAs in differential comparisons, yellow indicates N/A.

**Figure 4:**
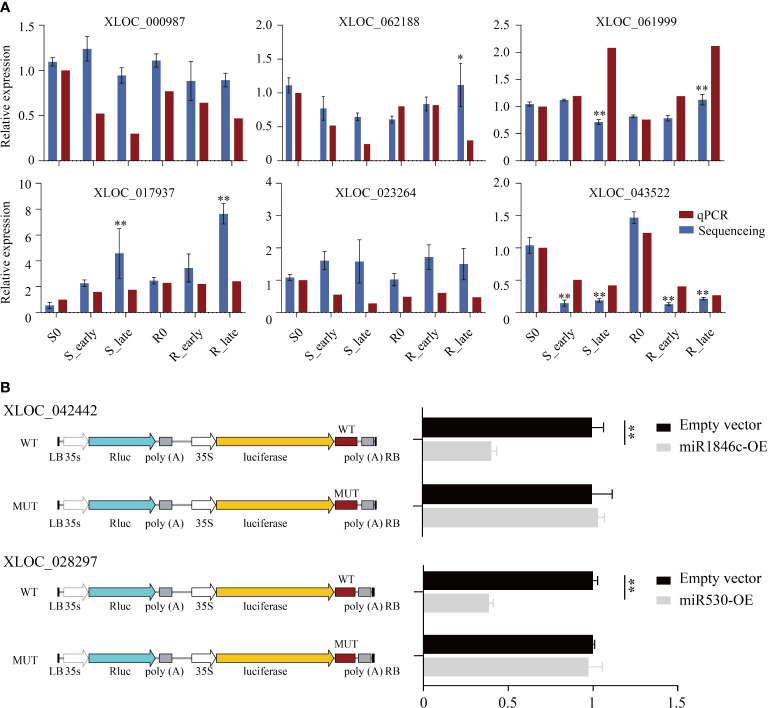
Detection of candidate lncRNAs expression levels and validation of target miRNAs. **(A)** DElncRNAs validated by qRT-PCR. **(B)** The luciferase reporter assay illustrates that candidate DElncRNAs could serve as a sponge for predicted target DEmiRNAs. Data are presented as the means ± SE of 3 biologically independent experiments. Data were analyzed by ANOVA and asterisks indicate statistically significant differences. *P < 0.05, **P < 0.01.

The lncRNA expression levels were compared between treatments, and the differentially expressed lncRNAs (DElncRNAs) exhibiting absolute value of log_2_FC ≥ 1 and *P* < 0.05 among the 6 treatment groups are shown in **Figure 3A**. The total number of DElncRNAs in group S (S_early/S_0 and S_late/S_0, 190) was higher than that of group R (R_early/R_0 and R_late/R_0, 108). During the late stage, the number of DElncRNAs in group S (S_late/S_0, 152) was 4 times greater than during the early stage (S_early/S_0, 38). The number of early and late DElncRNAs in group R was similar (R_early/R_0, 40; R_late/R_0, 68). There were 303 DElncRNAs identified in the three comparison groups among different varieties (R0/S0, R_early/S_early, and R_late/S_late) (**Figure 3A**), and the chromosomal distribution of the DElncRNAs was evaluated (**Figure 3B**). In order to identify lncRNAs related to BPH resistance in rice, we analyzed these DElncRNAs with a Venn diagram (**Figure 3C**). Overall, 60 DElncRNAs exhibited differential expression in R and S after BPH feeding (only in R_early/S_early and R_late/S_late). Thirty-two DElncRNAs were identified in R_early/R_0 which were absent in S_early/S_0 and 32 DElncRNAs were identified in R_late/R_0 which were absent in S_late/S_0. The expression profiles of these DElncRNAs were found for some modules (**Figure 3D**; **Supplementary Tables 2**, **3**). These results indicate that susceptible and resistant plants contained similar numbers of BPH-responsive lncRNAs during the early stage (38 in S_early/S_0 and 40 in R_early/R_0), although only 8 were identified in both groups (**Figure 3C**). Conversely, susceptible plants contained a greater number of BPH-responsive lncRNAs than resistant plants during the late stage (**Figures 3A, C**). That is, the two genotypes responded to BPH herbivory in entirely different manners. Subsequently, qRT-PCR was used to study the expression levels of these 6 DElncRNAs (**Figure 4A**). The data were consistent with the sequencing results, which confirmed that the results were reliable and could be used for intensive studies. Overall, the results confirmed that the numbers and types of BPH-responsive lncRNAs were different between resistant and susceptible plants, suggesting that lncRNAs are involved in the response of rice to BPH herbivory.

### Analysis of DElncRNA function via ceRNA regulatory networks in response to BPH feeding

In plants, lncRNAs indirectly regulate mRNA expression through their regulation of miRNA (Bartel, 2009; Thomas et al., 2010). By combining the psRNA target tool results with the miRNA and mRNA high-throughput sequencing data (Tan et al., 2020), we predicted the presence of 15, 30, 20, and 83 DElncRNAs binding to 15, 35, 35, and 87 differentially expressed miRNAs (DEmiRNAs) in R_early/R0, R_late/R0, S_early/S0, and S_late/S0, respectively (**Supplementary Figure 1**; **Supplementary Tables 4**–**7**). These DElncRNAs may affect the expression of their target differentially expressed mRNAs (DEmRNAs) (105, 242, 295, and 934 in R_early/R0, R_late/R0, S_early/S0, and S_late/S0, respectively) by sponging their corresponding DEmiRNAs (**Supplementary Tables 4**–**7**). The DElncRNAs, DEmiRNAs, and DEmRNAs in the ceRNA network were compared using Venn diagrams (**Supplementary Figure 1**).

To validate whether these DElncRNAs affected the expression of their corresponding DEmiRNAs, two DElncRNAs (XLOC_042442 and XLOC_028297) and their target DEmiRNAs (miR1846c and miR530) were selected for luciferase reporter assay verification. First, we constructed XLOC_042442 and XLOC_028297 WT or MUT (predicted miRNA binding sites) luciferase plasmids in the pGreenII 0800-miRNA dual luciferase reporter vector, and luciferase activity was evaluated after co-transfection of miRNA-containing (miR1846c and miR530, respectively) and luciferase plasmids. In rice protoplasts, we observed that overexpression of miR1846c and miR530 (miR1846c-OE and miR530-OE) reduced the luciferase activity significantly. At the same time, miRNA binding site mutations reversed the luciferase activity, suggesting that XLOC_042442 and XLOC_028297 function as ceRNAs by sponging miR1846c and miR530, respectively (**Figure 4B**).

The potential regulatory roles of the DElncRNAs via the ceRNA network were predicted by analyzing the functions of their target DEmRNAs through GO and KEGG pathway analysis (**Supplementary Figures 2**, **3**). The GO annotations (*P* < 0.05) of the four groups of target DEmRNAs contained multiple biological processes, cellular components, and molecular functions (**Supplementary Figure 2**). In BPH6G at the early feeding stage (R_early/R0), these GO terms were most significantly enriched in DNA replication initiation, plastid inner membrane, and syn-pimara-7,15-diene synthase activity. In NIP at the early feeding stage (S_early/S0), these GO terms were most significantly enriched in plant-type cell wall biogenesis, plasma membrane, and cellulose synthase activity. In BPH6G at the late feeding stage (R_late/R0), these GO terms were most significantly enriched in hydrogen peroxide catabolic process, integral component of membrane, and transmembrane transporter activity. In NIP at the late feeding stage (S_late/S0), these GO terms were most significantly enriched in regulation of hormone levels, plasma membrane, and ATP binding. KEGG pathway analysis was performed on the four groups of DEmRNAs, which showed that DNA replication and metabolic pathways were the most significantly enriched pathways (**Supplementary Figure 3**).

### Analysis of circRNA characteristics and response to BPH

**Figure 5:**
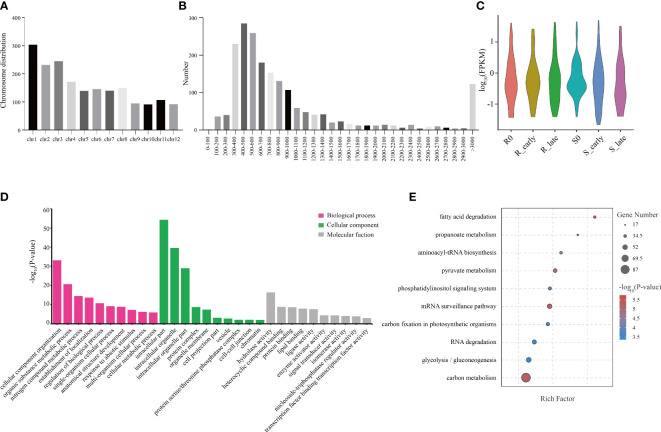
Distribution of circRNAs and GO and KEGG analyses of source genes. **(A)** Chromosomal distribution of circRNAs. **(B)** Lengths of circRNAs. **(C)** FPKM distribution of circRNAs in six groups. **(D)** GO enrichment analysis of circRNA source genes. **(E)** KEGG pathway enrichment analysis of circRNA source genes.

In this study, we identified a total of 1914 circRNAs, and the genomic distribution of these circRNAs is shown in **Figure 5A**. Different chromosomes contained different numbers of circRNAs, with chromosome 1 (Chr 1) containing the greatest number (**Figure 5A**). In terms of length, the majority of circRNAs were between 300-600 bp (**Figure 5B**). The circRNA expression levels among the 6 treatment groups were visualized using violin plots (**Figure 5C**). GO annotation of the circRNA source genes showed that they were enriched in the following biological process terms: cellular component organization, organic substance metabolic process, and nitrogen compound metabolic process; the following cellular component terms: intracellular part, intracellular organelle, and intracellular organelle part; and the following molecular function terms: hydrolase activity, heterocyclic compound binding, and protein binding (**Figure 5D**). KEGG pathway analysis showed that fatty carbon metabolism, glycolysis/gluconeogenesis, RNA degradation, carbon fixation in photosynthetic organisms, and mRNA surveillance pathway were most significantly enriched (**Figure 5E**).

**Figure 6:**
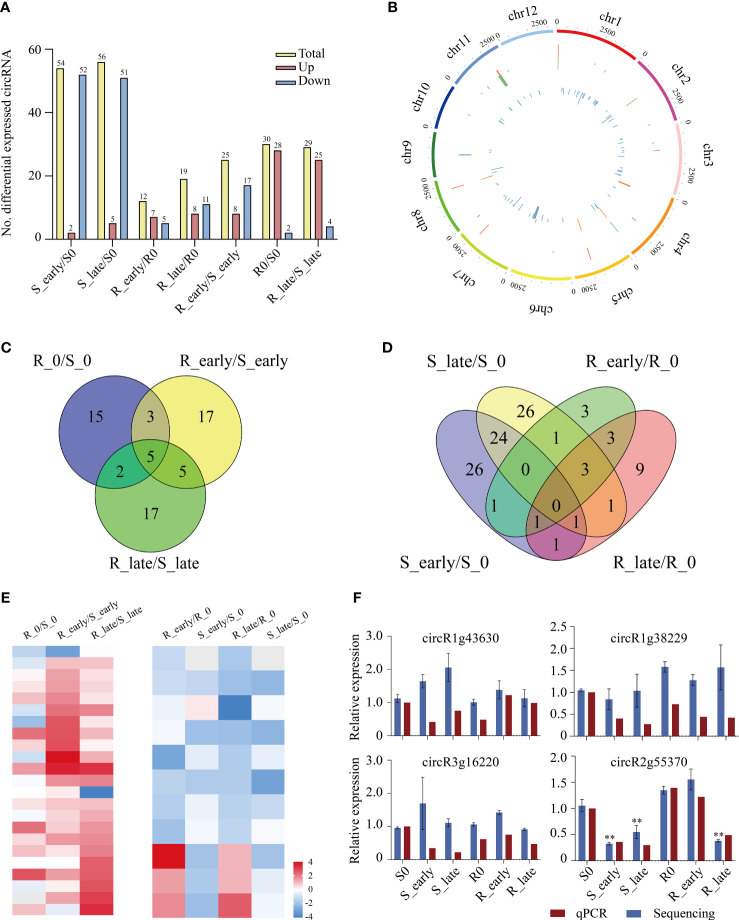
Differentially expressed circRNAs. **(A)** Number of DEcircRNAs up- or down-regulated in all comparisons. **(B)** Genomic distribution of all DEcircRNAs. The two circles (from outer to inner) represented the expression levels (log_10_FPKM) of DEcircRNAs in the resistant plants (red indicates increased expression, and green denotes decreased expression), DEcircRNAs in the susceptible plants (orange indicates increased expression, and blue denotes decreased expression), and fold change of the expression levels, respectively. **(C-D)** Venn diagrams of the unique and shared DEcircRNAs. **(E)** Heat map of the unique DEcircRNAs in differential comparisons. **(F)** Validation of candidate DEcircRNAs expression levels by qRT-PCR. circR1g43630, chr1_25000624_24999798_+826-LOC_Os01g43630; circR1g38229, chr1_21426198_21424262_+1936-LOC_Os01g38229; circR3g16220, chr3_8951244_8940833_-10411-LOC_Os03g16220; circR2G55370, chr2_33928616_33914366_+14250-LOC_Os02g55370. Data were analyzed by ANOVA and asterisks indicate statistically significant differences. **P < 0.01.

The circRNAs expression levels were compared between treatments, and we identified a total of 129 differentially expressed circRNAs (DEcircRNAs) exhibiting absolute value of log_2_FC ≥ 1 and *P* < 0.05 among the 6 treatment groups (**Figure 6A**). Group S (S_early/S_0 and S_late/S_0, 110) contained 3 times more DEcircRNAs than group R (R_early/R_0 and R_late/R_0, 31), and the DEcircRNAs in group S were primarily down-regulated. The numbers of DEcircRNAs in the early and late stages of group R were similar (R_early/R_0, 12; R_late/R_0, 19), and less than that in group S (S_early/S_0, 54; S_late/S_0, 56). The numbers of up- and down-regulated DEcircRNAs were similar in group R (7/5 in R_early/R0, 8/11 in R_late/R0). There were 84 DEcircRNAs contained in the three comparison groups among different varieties (R0/S0, R_early/S_early, and R_late/S_late) (**Figure 6A**). The distribution and expression of DEcircRNAs were also mapped to the chromosomes of the two materials (**Figure 6B**).

In order to identify circRNAs related to BPH resistance in rice, we analyzed the DEcircRNAs between and within groups of resistant and susceptible plants before and after BPH feeding using Venn diagrams (**Figures 6C, D**). A total of 39 DEcircRNAs exhibited differential expression between R and S after BPH feeding (only in R_early/S_early and R_late/S_late). Ten DEcircRNAs were identified in R_early/R_0 which were absent in S_early/S_0, and 14 DEcircRNAs were identified in R_late/R_0 which were absent in S_late/S_0. The expression patterns of these DEcircRNAs are shown as a heat map (**Figure 6E**; **Supplementary Tables 8**, **9**). The quantitative detection results were consistent with the sequencing data, which confirmed the reliability of the sequencing results (**Figure 6F**). Overall, these results confirmed that the numbers and types of BPH-responsive circRNAs were different between resistant and susceptible plants, suggesting that circRNAs are involved in the response of rice to BPH herbivory.

By combining the psRNA target tool results with the miRNA and mRNA high-throughput sequencing data (Tan et al., 2020), we predicted the existence of 8, 10, 19, and 26 DEcircRNAs binding to 42, 61, 29, and 114 DEmiRNAs in the R_early/R0, R_late/R0, S_early/S0, and S_late/S0 comparisons, respectively (**Supplementary Tables 10**–**13**). These DEcircRNAs may affect the expression of their target DEmRNAs (187, 401, 222, and 1090 in R_early/R0, R_late/R0, S_early/S0, and S_late/S0, respectively) by sponging their corresponding DEmiRNAs (**Supplementary Tables 10**–**13**). The DEcircRNAs, DEmiRNAs, and DEmRNAs in the ceRNA network were compared by Venn diagrams (**Supplementary Figure 1**).

The potential regulatory roles of the circRNAs via the ceRNA network were predicted by analyzing the functions of all the target DEmRNAs through GO and KEGG pathway analyses (**Supplementary Figures 4**, **5**). The GO annotations (*P* < 0.05) of the four groups of DEmRNAs are listed in **Supplementary Figure 3** and **Supplementary Tables 10**–**13**, and include a multitude of biological processes, cellular components, and molecular functions. In BPH6G at the early feeding stage (R_early/R0), these GO terms were most significantly enriched in cell wall biogenesis, photosystem II antenna complex, and kinase activity. In NIP at the early feeding stage (S_early/S0), these GO terms were most significantly enriched in plant-type cell wall biogenesis, plasma membrane, and cellulose synthase (UDP-forming) activity. In BPH6G at the late feeding stage (R_late/R0), these GO terms were most significantly enriched in metabolic process, integral component of membrane, and cellulose synthase (UDP-forming) activity. In NIP at the late feeding stage (S_late/S0), these GO terms were most significantly enriched in cell wall organization, plasma membrane, and ATP binding. KEGG pathway analysis was performed on the four groups of DEmRNAs, which showed that DNA replication and metabolic pathways were the most significantly enriched pathways (**Supplementary Figure 4**).

### Analysis of key ceRNA pathways responding to BPH infestation

**Figure 7:**
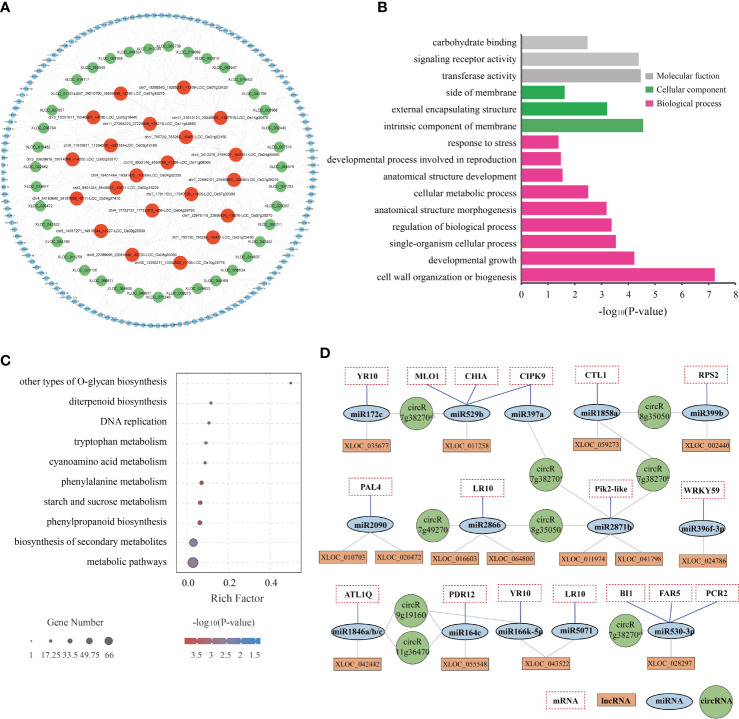
Regulatory network of potential BPH resistance-related lncRNAs and circRNAs. **(A)** CeRNA networks of potential BPH resistance-related DElncRNAs/DEcircRNAs-DEmiRNAs. Different colored circles indicate different non-coding RNA types: red represents DEcircRNAs, green represents DElncRNAs, and blue represents DEmiRNAs. **(B)** GO enrichment of 834 target DEmRNAs regulated by the ceRNA network. **(C)** KEGG enrichment of 834 target DEmRNAs regulated by the ceRNA network. **(D)** Core ceRNA networks of lncRNAs/circRNAs-miRNAs-mRNAs were significantly enriched in GO term “response to stimulus” (GO: 0006950). circR7g38270^a^, chr7_22980701_22946863_-33838-LOC_Os07g38270; circR7g38270^b^, chr7_22976110_22956434_-19676-LOC_Os07g38270; circR8g35050, chr8_22086695_22054445_-32250-LOC_Os08g35050; circR7g49270, chr7_29510790_29508498_+2292-LOC_Os07g49270; circR9g19160, chr9_11675871_11394316_+281555-LOC_Os09g19160; circR11g36470, chr11_21913121_20045605_-1867516-LOC_Os11g36470.

The lncRNA/circRNA-miRNA-mRNA interaction networks were predicted using psTarget software and consolidated using whole-transcriptome RNA sequencing data (**Figure 7A**; **Supplementary Table 14**). Specifically, the interaction network was established based on the relationship between the DElncRNAs/DEcircRNAs and DEmiRNAs and DEmRNAs. A total of 39 DElncRNAs were predicted to bind 50 DEmiRNAs and 381 target DEmRNAs, while 21 DEcircRNAs were predicted to bind 116 DEmiRNAs and 760 target DEmRNAs. The ceRNA network with 39 DElncRNAs, 21 DEcircRNAs, 133 DEmiRNAs, and 834 DEmRNAs is depicted in **Figure 7A** and **Supplementary Table 14**.

The potential regulatory roles of the ceRNA networks were analyzed by characterizing the function of 834 target DEmRNAs through GO enrichment and KEGG pathway analyses. GO annotation showed that the network was primarily enriched in the following biological processes: cell wall organization or biogenesis, developmental growth, and single-organism cellular process; the following cellular components: intrinsic component of membrane, external encapsulating structure, and side of membrane; and the following molecular functions: transferase activity, signaling receptor activity, and carbohydrate binding (**Figure 7B**). In addition, the network was found to be enriched in the following KEGG pathways: metabolic pathways, biosynthesis of secondary metabolites, phenylpropanoid biosynthesis, starch and sucrose metabolism, and phenylalanine metabolism (**Figure 7C**). These results illustrated that both metabolism- and response-related genes are regulated by the ceRNA network in both resistant and susceptible plants in response to BPH herbivory.

Notably, the GO term “response to stimulus” (GO: 0006950) was significantly enriched in 80 DEmRNAs (**Supplementary Table 14**). After consultation of relevant literature, several specific components of the lncRNA/circRNA-miRNA-mRNA interaction network were selected for further study: 6 DEcircRNAs, 15 DElncRNAs, 16 DEmiRNAs, and 17 DEmRNAs (**Figure 7D**). The lncRNAs XLOC_024786, XLOC_055548, and XLOC_043522, as well as the circRNAs circR9g19160 and circR11g36470, were predicted to combine with miR396f-3p, miR164c, and miR166k-5p. These three miRNA families have been described as regulators of rice innate immunity against fungi and bacteria (Raquel et al., 2018; Wang et al., 2018b; Li et al., 2019). In plants, *R* genes with the NB-ARC structure play vital roles in disease and pathogen resistance by recognizing specific effectors and inducing rapid and robust resistance responses (Jones and Dangl, 2006). In this study, we identified 6 NB-ARC domain-containing *R* proteins (MLO1-like, RPS2-like, RPM1-like, PIK2-like, RGA4-like (YP10), and RGA5-like (LP10)), which were negatively regulated by miR529b, miR399b, miR2886, miR2871b, miR396f-3p, and miR172c/miR166k-5p. These miRNAs were sponged by lncRNAs XLOC_011258, XLOC_002440, XLOC_016603/XLOC_064800, XLOC_011974/XLOC_041798, XLOC_024784, and XLOC_035677/XLOC_043522, respectively. Both *CTL1/BC15* and *CHIA*, which encode membrane-associated chitinase-like proteins, have been reported to be involved in rice resistance (Wu et al., 2012), and are negatively regulated by the XLOC_059273/circR8g35050-miR1858a and XLOC_011258/circR7g38270-miR529b modules, respectively. Additionally, *CIPK9*, *ATL1Q*, and *PDR12* have been reported to be involved in signal transduction and play significant roles in abiotic and biotic stress response (Guzmán, 2012; Kanwar et al., 2014; Nguyen et al., 2014). These genes were predicted to be the targets of miR397a, miR1846a/b/c, and miR164c, and these miRNAs were predicted to be regulated by circR7g38270, XLOC_042442, circR9g19160, circR11g36470, and XLOC_055548. Finally, *PAL4* has been associated with broad-spectrum disease resistance in rice (Tonnessen et al., 2015), and is negatively regulated by the XLOC_010703-miR2090 and XLOC_020472-miR2090 modules. These examples imply that ceRNAs might play important regulatory roles in the resistance of rice to BPH herbivory.

**Figure 8:**
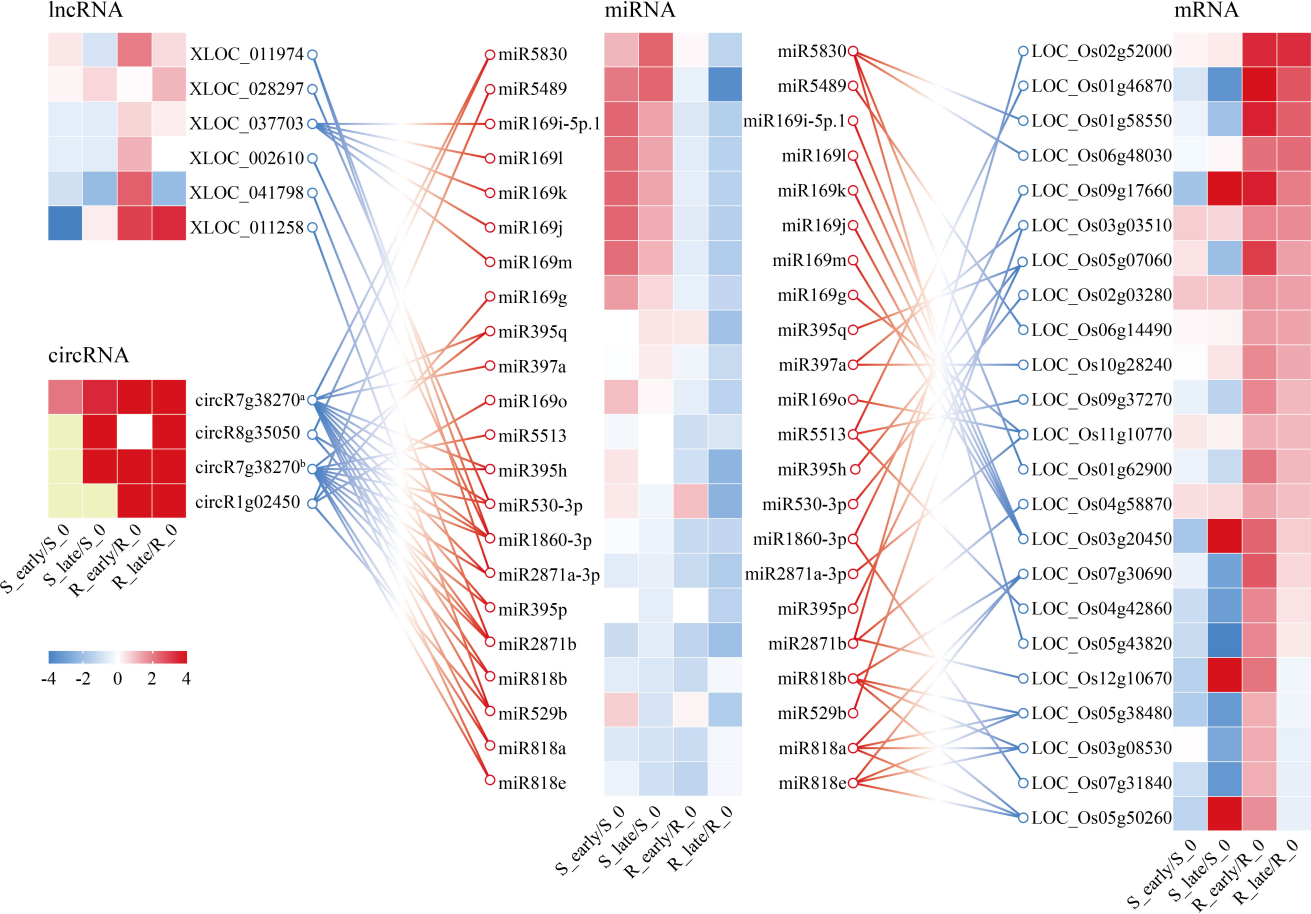
CeRNA networks of lncRNA/circRNA-miRNA-mRNA related to plant resistance. Yellow indicates N/A. circR7g38270^a^, chr7_22980701_22946863_-33838-LOC_Os07g38270; circR8g35050, chr8_22086695_22054445_-32250-LOC_Os08g35050; circR7g38270^b^, chr7_22976110_22956434_-19676-LOC_Os07g38270; circR1g02450, chr1_795720_785269_-10451-LOC_Os01g02450.

In a previous study, an integrated analysis of the miRNAs and target mRNA genes related to BPH resistance in *BPH6*-transgenic plants and NIP was performed (Tan et al., 2020). A network of 34 miRNAs corresponding to 42 target genes was identified, which were potentially related to BPH resistance (Tan et al., 2020). Meanwhile, considering our ceRNA results, we obtained a core ceRNA network consisting of 6 lncRNAs, 4 circRNAs, 23 miRNAs, and 24 mRNAs (**Figure 8**). This core ceRNA network might also play a vital role in the resistance of rice to BPH herbivory.

## Discussion

In rice, both lncRNAs and circRNAs have been reported to regulate plant growth and development, as well as the biotic and abiotic stress responses (Sanchita and Asif, 2020; Zhang et al., 2020). To date, several lncRNAs have been functionally characterized in rice. For example, *LDMAR* (*long-day-specific male fertility-associated RNA*) is a 1236bp photoperiod sensitive lncRNA which regulates male sterility in rice. The normal development of plant pollen under long-day conditions requires a high level of *LDMAR* expression (Fan et al., 2016). The expression of the lncRNA *IPS1* (*induced by phosphate starvation 1*) is altered under nitrogen and phosphorus starvation, suggesting that *IPS1* may regulate the balance of these macronutrients (Shin et al., 2018). Overexpression of the lncRNA *LAIR* (*leucine-rich repeat receptor kinase antisense intergenic RNA*) in rice increases grain yield and induces the expression of several leucine-rich repeat receptor kinase-coding genes (Wang et al., 2018a). Although there has been some research into plant circRNAs, currently-reported mechanisms of circRNA formation are largely based on bioinformatics analyses rather than convincing experimental evidence, which is still in the theoretical stage (Zhang et al., 2020). To the best of our knowledge, this is the first study to comprehensively identify and characterize BPH-responsive lncRNAs and circRNAs associated with ceRNA networks in rice. Therefore, the results presented here further our understanding of non-coding RNA regulation of the rice response to BPH infestation.

In this study, we identified BPH-responsive lncRNAs and circRNAs, and their differential expression patterns were analyzed in *BPH6*-transgenic and NIP rice plants. Our genome-wide analysis of lncRNA and circRNA expression showed that susceptible NIP plants contained greater numbers of both DElncRNAs and DEcircRNAs than resistant BPH6-transgenic plants, and that the majority of these DElncRNAs and DEcircRNAs were down-regulated in NIP plants. The functions of these lncRNAs and circRNAs via the ceRNA network were also predicted by GO and KEGG analyses of target mRNAs (**Supplementary Figures 2**–**5**). The GO and KEGG pathway results differed between the resistant and susceptible varieties at both the early and late feeding stages (**Supplementary Figures 2**–**5**). Our results suggest that the BPH-responsive lncRNAs and circRNAs in *BPH6G* and NIP were involved in different ceRNA pathways.

The interactions between different RNAs were predicted based on ceRNA theory, and multiple BPH-resistance related networks were identified and included 39 lncRNAs, 21 DEcircRNAs, 133 DEmiRNAs, and 834 DEmRNAs (**Figure 7A**; **Supplementary Table 14**). The functions of the ceRNA networks were then explored through GO and KEGG pathway analyses (**Figures 7B, C**). Among the ceRNA networks, 80 DEmRNAs were predicted to be involved in the response to stress (GO: 0006950) (**Supplemental Table 15**). A core ceRNA network of these DEmRNAs is shown in **Figure 7D**. Among them, OsmiR396 has been identified as a negative regulator of rice innate immunity against *Magnaporthe oryzae* by silencing multiple growth-regulating factors (*OsGRFs*) in rice (Chandran et al., 2019). Overexpression of OsmiR396 makes rice susceptible to blast, while suppression of OsmiR396 makes rice resistant to blast and improves yield (Chandran et al., 2019). In addition, overexpression of OsmiR396f resulted in enhanced immunity to *Dickeya zeae* by suppressing the target gene *OsGRF* (Li et al., 2019). A previous study showed that OsmiR396 also acts as a negative regulator of BPH resistance via the OsmiR396-*OsGRF8*-*OsF3H*-flavonoid pathway (Dai et al., 2019). Our results showed that OsmiR396f-3p, which targets the *OsWRKY59* transcription factor, is regulated by lncRNA XLOC_024786. Hence, we speculate that the XLOC_024786-OsmiR396f-3p-*OsWRKY59* module may play a vital role in rice resistance to BPH herbivory (**Figure 7D**). OsmiR164a has been reported to negatively regulate rice immunity to *M. oryzae* and *Xanthomonas oryzae* pv. *oryzae* by regulating the *OsNAC60* transcription factor (Wang et al., 2018b; Jia et al., 2020). We also found that lncRNA XLOC_055549 could sponge miR164a, suggesting that the XLOC_055549-OsmiR164a module may be a key regulator of BPH resistance (**Figure 7D**). The miR166 family is highly conserved (Kumar et al., 2022). The miR166 family members OsmiR166k and OsmiR166h have been shown to function as positive regulators of defense against *M. oryzae* and *Fusarium fujikuroi* by targeting the *ethylene insensitive 2* (*EIN 2*) gene via cross-regulation (Raquel et al., 2018). This study demonstrated that lncRNA XLOC_043522 negatively regulates the expression of miR166k-5p, thus up-regulating the expression of the *R* gene *YR10* (**Figure 7D**). The ceRNA networks identified in this study are likely to play important roles in rice resistance to BPH. By integrating and analyzing the results of our ceRNA study, alongside the results of Tan et al.’s miRNA and target mRNA study, a core ceRNA network containing lncRNAs, circRNAs, miRNAs, and mRNAs was produced (**Figure 8**) (Tan et al., 2020). These results provide evidence of novel regulatory mechanisms underlying rice BPH resistance. Our future research will focus on elucidating of the BPH-responsive ceRNA network in rice, which will greatly increase our knowledge of plant resistance to insect damage.

The rice-BPH system is considered an excellent model for the study of plant-insect interaction and co-evolution (Jing et al., 2017). Xiao et al. provided evidence that lncRNAs might play important roles in the high fecundity and virulence adaptation of BPH (Xiao et al., 2015). A previous study has reported the differential expression of lncRNAs between two virulent BPH populations, including susceptible (TN1) and resistant (YHY15) rice planthopper varieties (Zha et al., 2022). In total, 157 differentially expressed lncRNAs and 675 differentially expressed mRNAs were found to be involved in BPH adaptation to rice resistance (Zha et al., 2022). Here, we studied the differential expression of lncRNAs and circRNAs in rice before and after BPH herbivory. Our analysis indicated that both lncRNAs and circRNAs play important roles in rice resistance to BPH. These results suggest the presence of mutual regulatory relationships between rice and BPH at the non-coding RNA level, and provide a basis for further studies of the molecular mechanism underlying co-evolution between rice and BPH.

## Data availability statement

The datasets presented in this study can be found in online repositories. The names of the repository/repositories and accession number(s) can be found below: https://www.ncbi.nlm.nih.gov/, GSE123148.

## Author contributions

AY, BD, and YW conceived and designed the experiments. YW, WZ, and DQ performed the experiments and analyzed the data. YW wrote the paper. JG developed the BPH6G line. GL, CL, BW, SL, JC, LH, and SS contributed reagents, materials, and analysis tools. LH, LZ, and ZZ revised the paper. YW, WZ, and DQ contributed equally to this paper. All authors contributed to the article and approved the submitted version.

## Supplementary material

Supplementary Figure 1 Venn diagrams of the DElncRNAs, DEcircRNAs, DEmiRNAs, and DEmRNAs in the ceRNA network. **(A–C)**. Venn diagrams of DElncRNAs, DEmiRNAs, and DEmRNAs in the ceRNA network of lncRNA-miRNA-mRNA. **(D–F).** Venn diagrams of DEcircRNAs, DEmiRNAs, and DEmRNAs in the ceRNA network of circRNA-miRNA-mRNA.

Supplementary Figure 2 GO enrichment of the target genes of DElncRNAs via the ceRNA network at the early and late feeding stages in susceptible and resistant plants.

Supplementary Figure 3 KEGG pathway analysis of the target genes of DElncRNAs via the ceRNA network at the early and late feeding stages in susceptible and resistant plants. The red color represents the pathways with *P* < 0.05.

Supplementary Figure 4 GO enrichment of the target genes of DEcircRNAs via the ceRNA network at the early and late feeding stages in susceptible and resistant plants.

Supplementary Figure 5 KEGG pathway analysis of the target genes of DEcircRNAs via the ceRNA network at the early and late feeding stages in susceptible and resistant plants. The red color represents the pathways with *P* < 0.05.
