# Supplementary figures and images for: Comprehensive identification and characterization of lncRNAs and circRNAs reveal potential brown planthopper-responsive ceRNA networks in rice
Source: Front Plant Sci. 2023 Aug 10;14:1242089. doi: 10.3389/fpls.2023.1242089 (PMC10457010; doi:10.3389/fpls.2023.1242089)

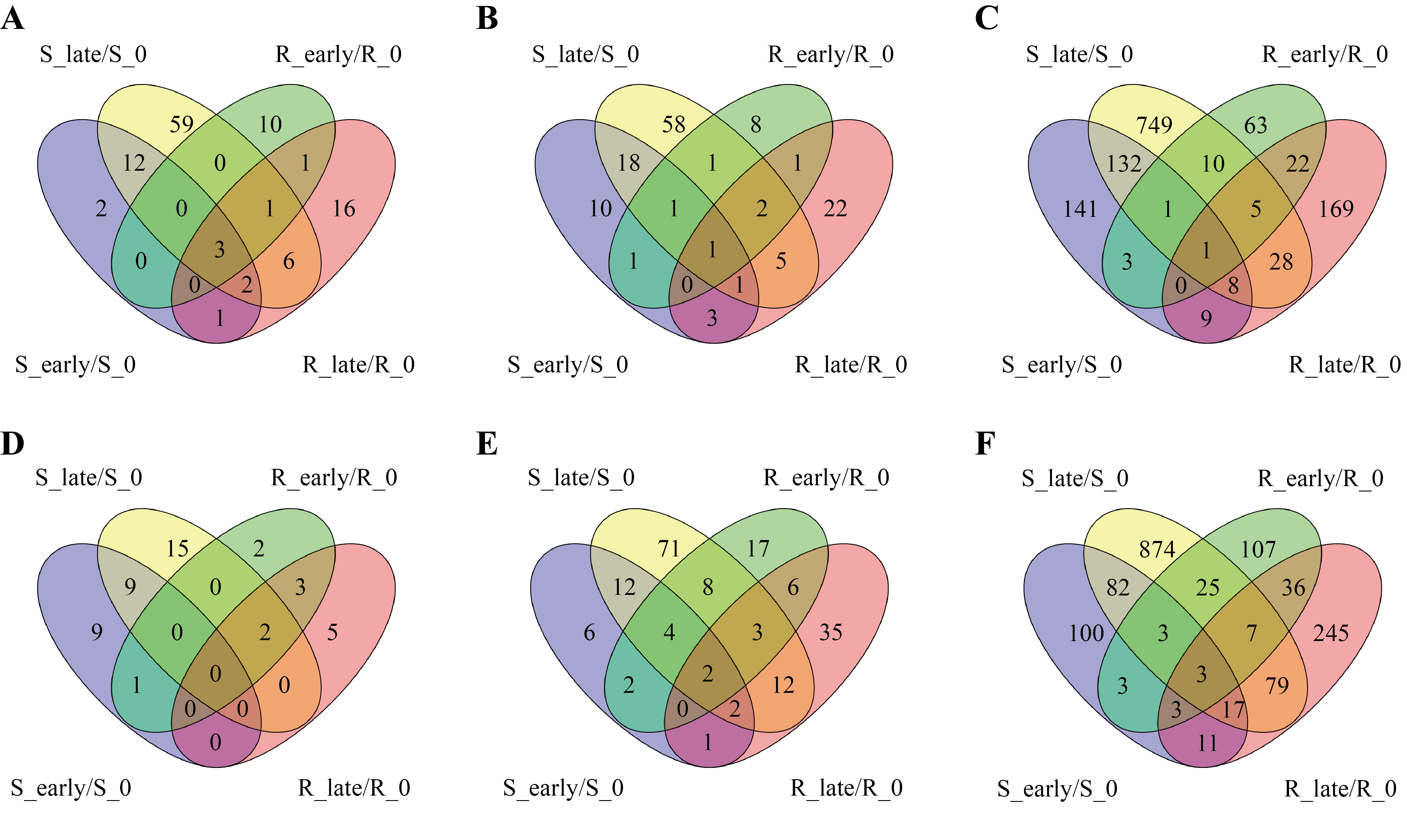

Supplement: Supplementary Figure 1 — Venn diagrams of the DElncRNAs, DEcircRNAs, DEmiRNAs, and DEmRNAs in the ceRNA network. (A–C). Venn diagrams of DElncRNAs, DEmiRNAs, and DEmRNAs in the ceRNA network of lncRNA-miRNA-mRNA. (D–F). Venn diagrams of DEcircRNAs, DEmiRNAs, and DEmRNAs in the ceRNA network of circRNA-miRNA-mRNA. [file Image_1.jpeg]

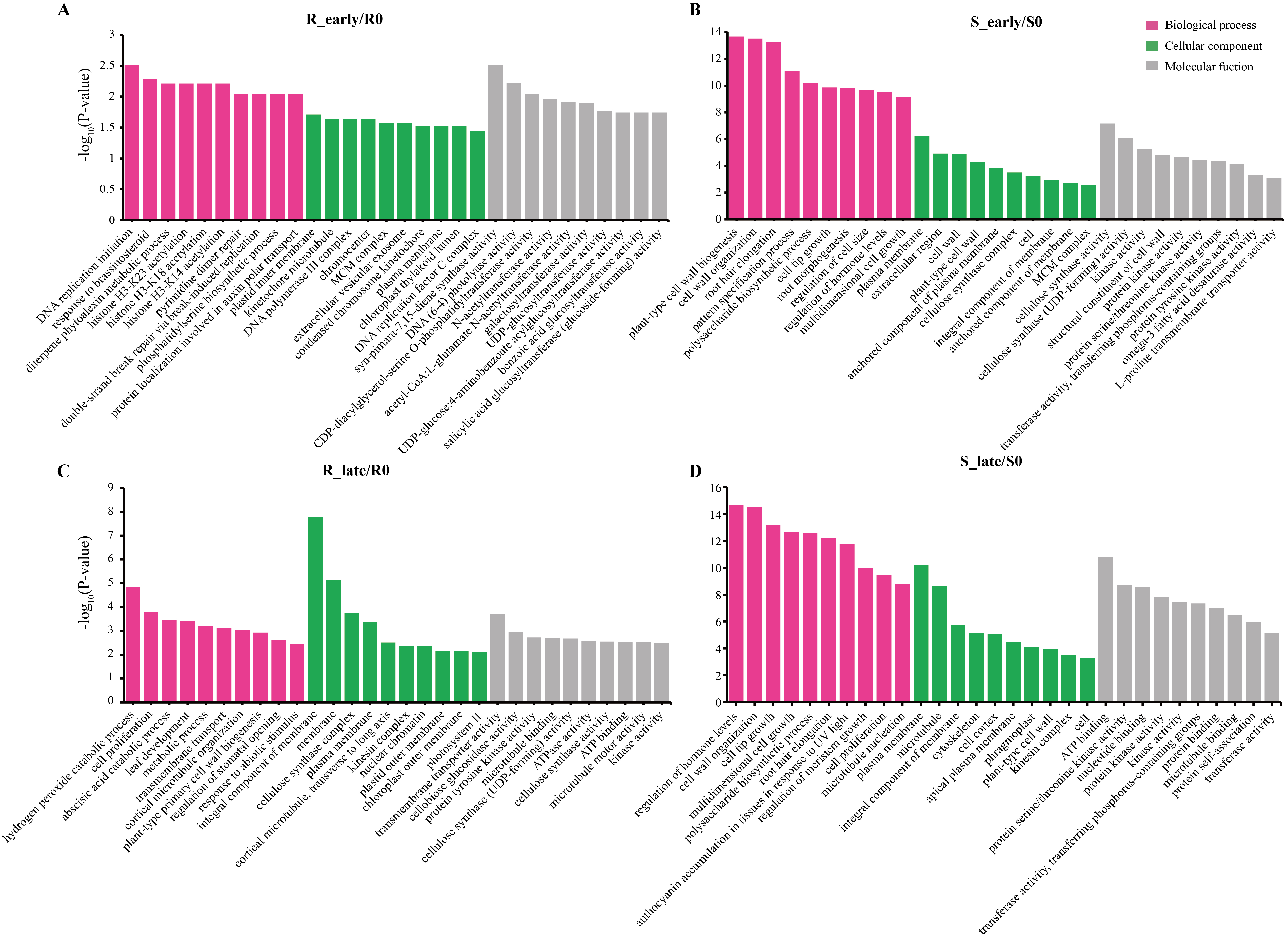

Supplement: Supplementary Figure 2 — GO enrichment of the target genes of DElncRNAs via the ceRNA network at the early and late feeding stages in susceptible and resistant plants. [file Image_2.jpeg]

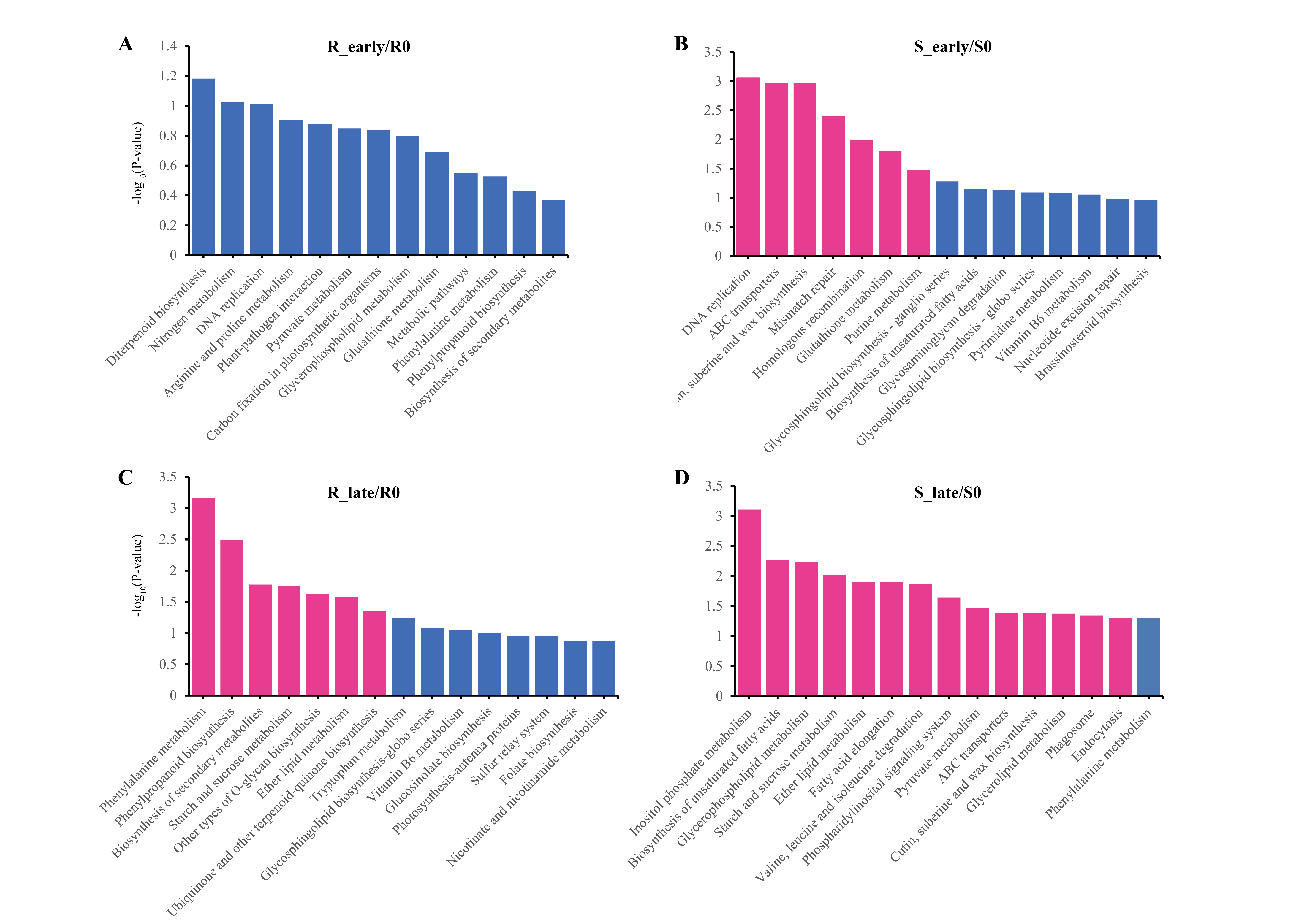

Supplement: Supplementary Figure 3 — KEGG pathway analysis of the target genes of DElncRNAs via the ceRNA network at the early and late feeding stages in susceptible and resistant plants. The red color represents the pathways with P < 0.05. [file Image_3.jpeg]

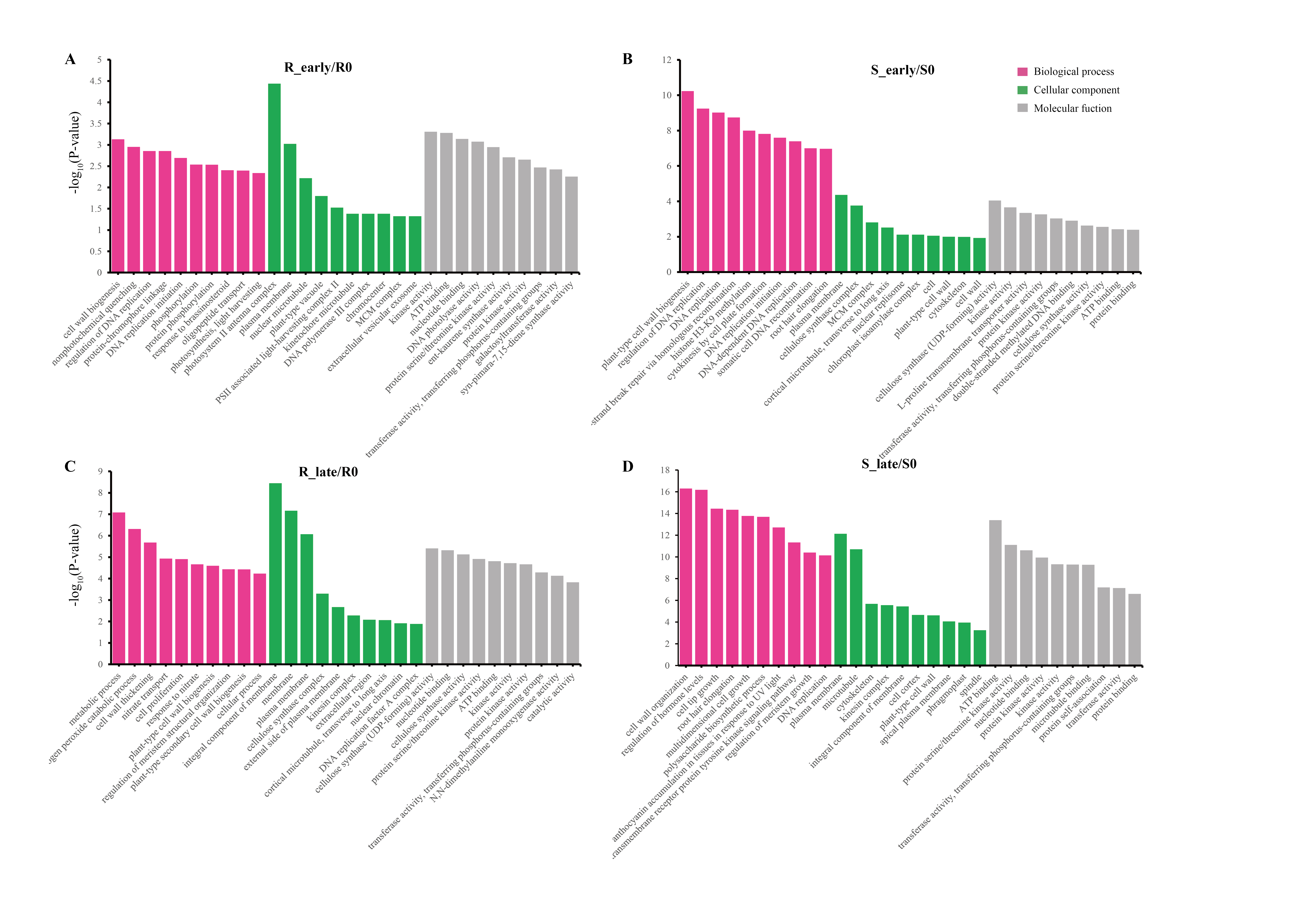

Supplement: Supplementary Figure 4 — GO enrichment of the target genes of DEcircRNAs via the ceRNA network at the early and late feeding stages in susceptible and resistant plants. [file Image_4.jpeg]

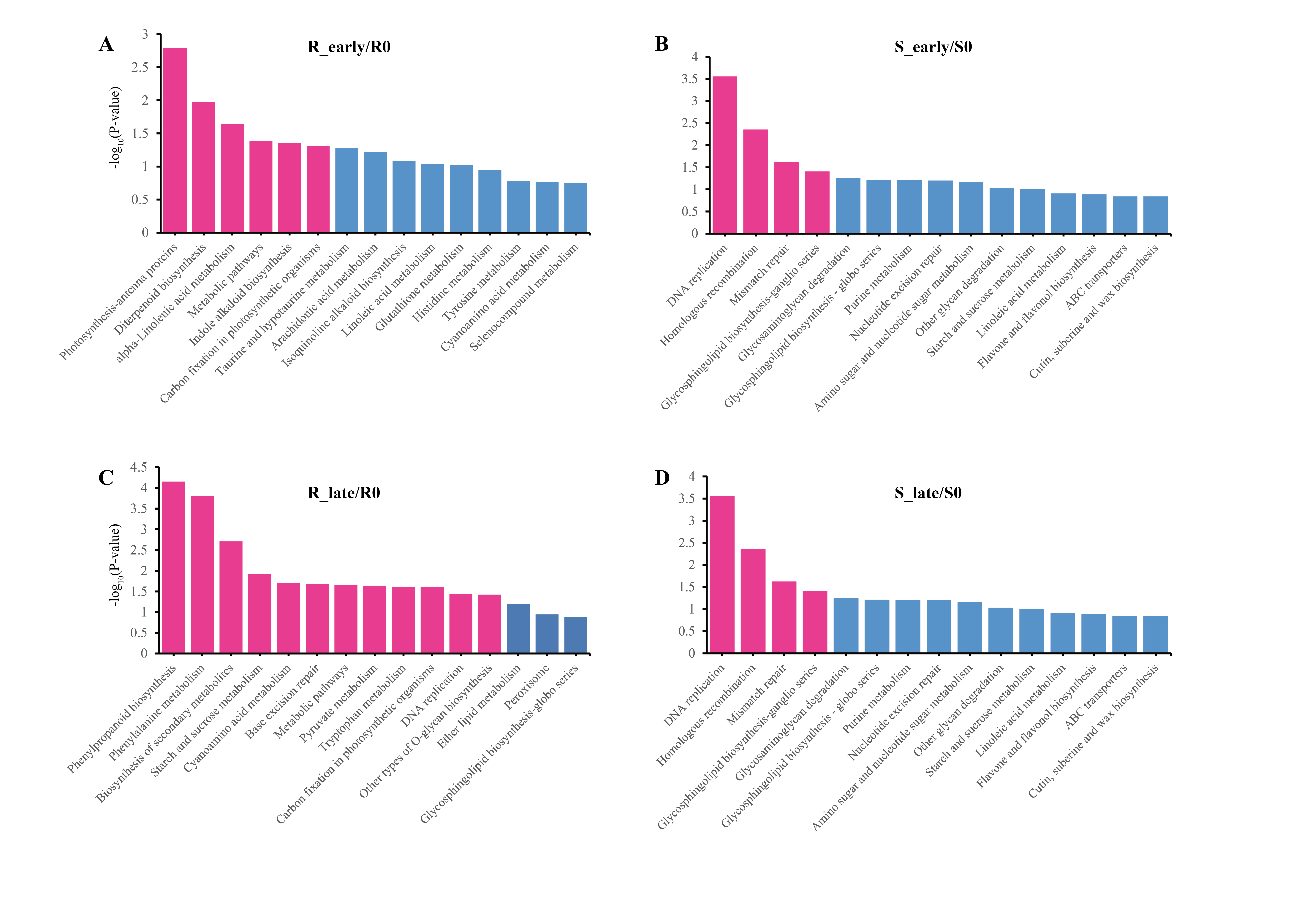

Supplement: Supplementary Figure 5 — KEGG pathway analysis of the target genes of DEcircRNAs via the ceRNA network at the early and late feeding stages in susceptible and resistant plants. The red color represents the pathways with P < 0.05. [file Image_5.jpeg]
